# Acute responses of strength-related gene expressions to maximum strength and force sense acuity

**DOI:** 10.55730/1300-0144.5775

**Published:** 2023-12-04

**Authors:** Muammer ALTUN, Erdal BALCAN, Sevinç BATIR, Mehmet Hilmi GÖKMEN, Şule ÖZGÜNEŞ, Zübeyde ÖZTEL

**Affiliations:** 1 Department of Training Education, Faculty of Sport Sciences, Manisa Celal Bayar University, Manisa, Turkiye; 2 Department of Biology, Molecular Biology Section, Faculty of Science and Art, Manisa Celal Bayar University, Manisa, Turkiye

**Keywords:** Proprioception, gene expression, qRT-PCR, muscle, strength

## Abstract

**Background/aim:**

Although high muscle strength worsens the sense of force, it is unknown whether there is a relationship between this deterioration and the underlying molecular mechanisms. This study examined the relationship between decreased force sense (FS) acuity and strength-related gene expressions.

**Materials and methods:**

Maximal voluntary isometric contraction (MVIC) and FS (50% MVIC) tests were performed on the knee joints of twenty-two subjects. The expression analyses were evaluated by qRT-PCR in blood samples taken before, after MVIC, after 50% MVIC, and 15 min after the test.

**Results:**

MVIC and FS error values were significantly correlated with each other (r = .659, p = .001). The qRT-PCR analyses demonstrated that the expressed mRNAs of the interleukin 6 (IL-6), alpha-actinin 3 (ACTN3), angiotensin-converting enzyme (ACE), brain-derived neurotrophic factor (BDNF), and ciliary neurotrophic factor receptor (CNTFR) genes dramatically increased until 50% MVIC and subsequently decreased 15 min after the exercise (p < .05). The muscle-specific creatine kinase (CKMM), myosin light chain kinase (MLCK), and G-protein β3 subunit (GNB3) genes reached their peak expression levels 30 min after MVIC (p < .05). ACE and ACTN3 gene expression increased significantly in parallel with the increased FS error (p < .05). These gene expression fluctuations observed at 50% MVIC and after the rest could be related to changes in cellular metabolism leading to fatigue.

**Conclusion:**

The time points of gene expression levels during exercise need to be considered. The force acuity of those whose maximal force develops too much may deteriorate.

## 1. Introduction

Many sporting skills require accurate and well-controlled muscle strength with good proprioception in the knee joints [1]. Knee proprioception is usually examined by assessing force sense (FS) and joint position sense. Although recently published research revealed that high muscle strength worsens FS [2,3], which molecular mechanisms underlying promoting this deterioration remain an open question. A series of genetic analyses suggested that skeletal muscle strength is associated with various genes such as an angiotensin-converting enzyme (ACE), alpha-actinin 3 (ACTN3), ciliary neurotrophic factor (CNTF), vitamin D receptor, and myostatin-related genes [4].

The polymorphisms of interleukin 6 (IL-6), ACTN3, ACE, muscle-specific creatine kinase (CKMM), myosin light chain kinase (MLCK), brain-derived neurotrophic factor (BDNF), ciliary neurotrophic factor receptor (CNTFR), and G-protein *β_3_ subunit* (GNB3) genes are effective in maximal force reproduction and nerve conduction. However, their relationship to the sensation of force at submaximal loads has never been investigated. IL-6 is a cytokine that has a pleiotropic effect. Apart from its role in the immune system, it is also essential for muscle recovery and athletic performance [5]. Another essential gene that affects strength and muscle size is the ACTN3 coding alpha actinin-3 protein in fast-twitch skeletal muscle fibers responsible for high-intensity exercises. The ACTN3 determines high-speed contractions and high-power production [6]. ACE is highly associated with the rennin-Angiotensin-Aldosteron system involved in blood pressure regulation and maintaining salt and fluid balance. Previous reports noted that the deletion (D) allele of ACE might be highly associated with the ability and discipline of elite athletes [7]. The role of CKMM in the energy homeostasis of muscle cells is crucial, and the activity of the CKMM gene is significantly higher in type II muscle fiber (fast twitches). The MLCK gene plays an important role in strength development, especially in fast-contracting muscles [8]. BDNF regulates neurogenesis, neural transmission speed, or nerve conduction velocity and positively affects brain health during regular exercise [9]. CFTR is expressed in human skeletal muscle cells and is mainly associated with muscle strength and type 2 fiber type. The CNTFR gene is associated with muscle strength and sportive performance [10]. The GNB3 gene is responsible for the transduction of cell signals between intracellular effectors and is a candidate to explain human variability and exercise phenotypes [11].

In the present study, to test whether genetic factors’ oscillations influence the human body’s physical performance, we evaluated the statistical relationship between muscle strength and candidate genes that can be responsible for force sense during the different muscle strength conditions. This study examines whether the decrease in force sense acuity relates to gene expressions. No studies show how long after exercising these genes reach high levels in the blood. Therefore, this study will reveal their acute expression responses.

## 2. Materials and methods

### 2.1 Participants

This study was approved by the Manisa Celal Bayar University Health Sciences Ethics Committee for non-interventional researches (15.01.2020–20.478.486/223). All participants agreed to volunteer for the research by giving written and verbal details about the procedure and aims of the research. Personal data of the volunteers were secured with the “informed volunteer consent form”. Twenty-two physically active subjects (14 males and 8 females; age: 19.6 years, mass: 71.3 kg, and body fat ratios: 11.3%) with no history of knee pathology participated in the study voluntarily. These were healthy subjects who practiced recreational sports at least three days a week and were individual or team players. Participants were required to abstain from medications impacting nervous system function. Those with acute or chronic neuromuscular disease or orthopedic knee joint disorders were excluded. Informed volunteer participants did not carry out exhausting physical activities in the last 24 h before the study. Before breakfast, body composition was analyzed using the InBody 230 bioelectrical impedance analyzer (Biospace Ltd., Seoul, Korea) [12]. Participants’ activity levels (6.8) were assessed according to the Tegner Activity Scale [13].

### 2.2 Experimental approach to the problem

Prior to the test, participants completed a warm-up. They were familiarized with the test using different random target angles and low-force replication levels. Familiarization was provided by purposefully avoiding a 45°° angle with two trials for every condition.

Maximum voluntary isometric contraction (MVIC) and FS analysis were conducted on the Isoforce (TUR Therapietechnik GmbH, Berlin, Germany) isokinetic dynamometer. Previous studies proved this device is reliable for knee and other joint assessments [14]. Participants sat on the dynamometer with a hip and knee angle of 90°. The dominant leg was attached to the power arm, and the thigh was fixed to the seat. To determine limb dominance, participants were asked which leg they used to kick a ball. The dynamometer’s axis was aligned with the knee joint rotation axis (lateral femoral epicondyle). This sitting position is a valid position used in knee MVIC and FS tests and considered starting position as 0°for knee extension [14]. Since only one leg was measured, absolute error values were analyzed for MVIC and FS tests.

### 2.3 MVIC and FS tests

MVIC strength was tested at a 45-degree knee extension angle, with test times of 5 s, and was tested three times, with 5 min of rest between sets [15]. The highest torque from the three trials was analyzed, and from this peak torque, the target force for the FS test was calculated. In the literature, 50% of the MVIC is used for force reproduction measurement [16,17]. After 15 min of rest, using 50% MVIC as the target force value, the strength of the knee joint was assessed by visual feedback of the target force on the computer screen [15]. The participant maintained the test position isometrically for 5 s to identify that position. After two trials, participants closed their eyes and reproduced the target force. They sustained the FS test force reproduction for 5 s and confirmed their predictions. Participants attempted the tests twice and rested for 90 s between trials to prevent fatigue [18]. FS error values were calculated from the absolute difference between the targeted and reproduced forces. The lowest error score between the two trials were analyzed.

### 2.4 Blood sample

Before starting the tests and active warming, the first 2 mL blood sample was drawn from the forearm at rest. About half an h after the first sample, the second blood sample was taken 3 min after the maximal strength test. The third sample was taken approximately 20 min after the second and 3 min after the 50% load test. The last blood sample was taken 15 min after the tests were finished, and the third blood sample was taken. During this time, complete rest was made. After blood collection, 45 min before transferring to the laboratory. It was stored at 4 °C to prevent it from spoiling. Then it was stored at −80 °C until the genetic analysis.

### 2.5 RNA extraction and quantitative real-time pcr (qRT-PCR) analyses

Total RNA extraction was performed from 0.2 mL peripheral venous blood samples using the PureLink® RNA Mini Kit (Thermo Fisher Scientific, 12183555). cDNA synthesis from the RNA samples was performed using a High-Capacity cDNA Reverse Transcription Kit (Applied Biosystems™, 4368814) in the SensoQuest thermal cycler. RNA expression levels of the IL-6, ACTN-3, ACE, CKMM, MLCK, BDNF, CNTFR, and GNB3 genes were acquired from the extracted RNA samples using IL6F1 5’-CACAGACAGCCACTCACCTC-3’, IL6R1 5’-GCCTCTTTGCTGCTTTCACA-3’, ACTN3F15’-CGCAGGACATCAACACCAAG-3’, ACTN3R1 5’-CGGAGCCTCTCGTTTACCTG-3’, ACEF1 5’-GCCAACCACACCCTGAAGTA-3’, ACER1 5’-TGCCCGTTCTAGGTCCTGAA-3’, CKMMF15’-TTAAGAAAGCTGGCCACCCC-3’, CKMMR15’-GGATCTCCTCGAACTTGGGG-3’, MLCKF15’-CCCGAGGTTGTCTGGTTCAA-3’, MLCKR15’-ATCGTCATCCCCGCAAACAT-3’, BDNFF1 5’-TCAAGTGCCTTTGGAGCCTC-3’, BDNFR15’-TACTGTCACACACGCTCAGC-3’, CNTFRF15’-TGACGTGGCGGGTAAATGG-3’, CNTFRR15’-GTGGAAGCAGGCGTAGAGG-3’, GNB3F1 5’-CTTCCTGCCGCTTGTTTGAC-3’, GNB3R1 5’-TTGAAGTCGTCGTAGCCAGC-3’ primers and SYBR Green Mix Kit (Genemark Bio). A separately prepared mixture of targeted genes primers and SYBR Green Mix were analyzed in the Rotor-Gene Q (Qiagen, Hilden, Germany). Normalizing of the target genes expression changes was performed according to the hypoxanthine phosphoribosyl transferase (HPRT1) (HPRT1F15’-CGTCTTGCTCGAGATGTGAT-3’, HPRT1R15’-TTCAGTGCTTTGATGTAATCCAG-3’) housekeeping gene expression values. The related forward and reverse primers were synthesized by Eurofins Scientific company. qRT-PCR cycling conditions started with the initial activation/denaturation stage of 95 °C (5 min), followed by 40 cycles of denaturation at 95 °C (15 s), combined annealing/extension 60 °C (60 s). The 2^−ΔΔCT^ method was used to calculate the relative changes in gene expression.

### 2.6 Statistical analyses

All data were analyzed using the SPSS 20.0 (SPSS Inc., Chicago, IL) package program at α = .05 significance level. We first investigated whether gene expression data were distributed normally via the Kolmogorov-Smirnov test. As the results of the Kolmogorov-Smirnov test illustrated that the data does not fit the normal distribution, we decided to implement nonparametric statistical technics. To explore whether gene expressions may vary due to load conditions, repeated measures of ANOVA were carried out. To see the significant main effect of load, multiple comparisons were conducted with Tukey correction. Finally, Pearson correlation coefficients were calculated to examine the relationship between MVIC and FS (50% MVIC) variables.

## 3. Results

Participants’ mean MVIC and absolute error scores were 53.65 ± 15.06 N and 3.83 ± 4.05 N for 45^o^ knee extensions, respectively. MVIC and FS error values were significantly correlated with each other’s (r = .659, p = .001). The correlation between MVIC peak torque and FS absolute error values showed that those with higher maximal isometric strength had more force sensitivity errors.

### 3.1 Tables and figures

**Figure 1:**
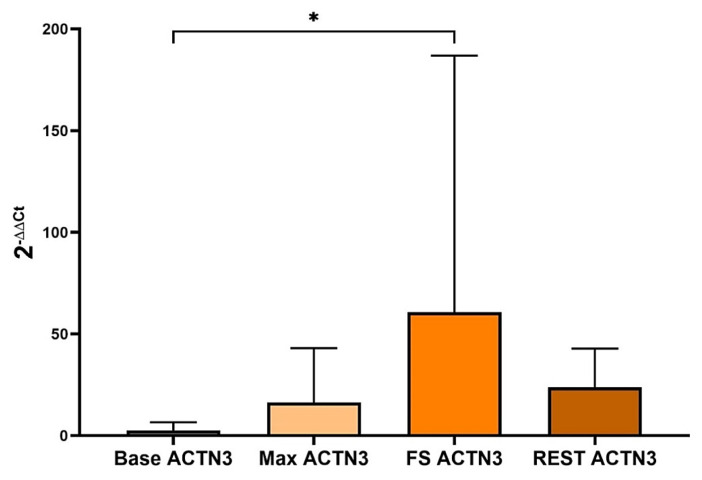
After 50% MVIC, the transcript of the ACTN is significantly increased when compared to the baseline (*p < .05; 60.8 ± 126.1).

The ACTN gene was significantly increased after 50% MVIC compared to baseline (p < .05; 60.8 ± 126.1). However, during the maximum performance or after the resting period, no significant increase was observed in our experiments. This data suggests that the expression of the ACTN transcript starts to increase during MVIC and reaches to maximum level after 50% MVIC (Figure 1).

**Figure 2:**
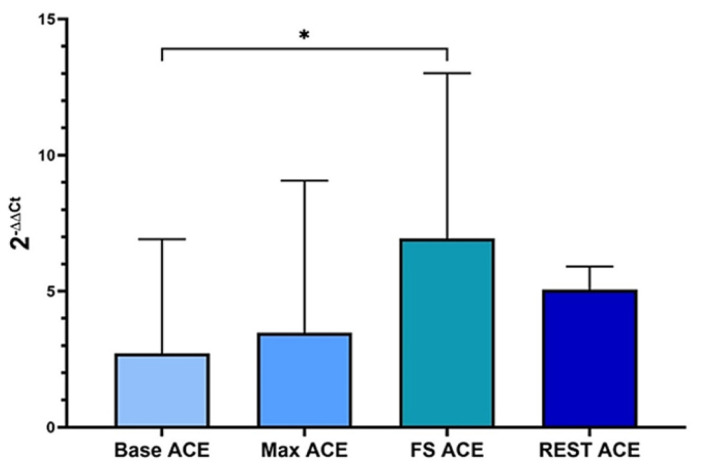
The ACE levels transiently increased until after 50% MVIC, which was significantly different at this condition as compared to the baseline (*p < .05) and decreased again after rest.

Although the ACE level steadily increased throughout tests, it decreased after the resting condition, as seen in Figure 2. The maximal expression level of the ACE was seen after 50% MVIC (p < .05, 6.94 ± 6.08). The result of the repeated measure of ANOVA illustrated that the ACTN3 values did not significantly differ among conditions.

**Figure 3:**
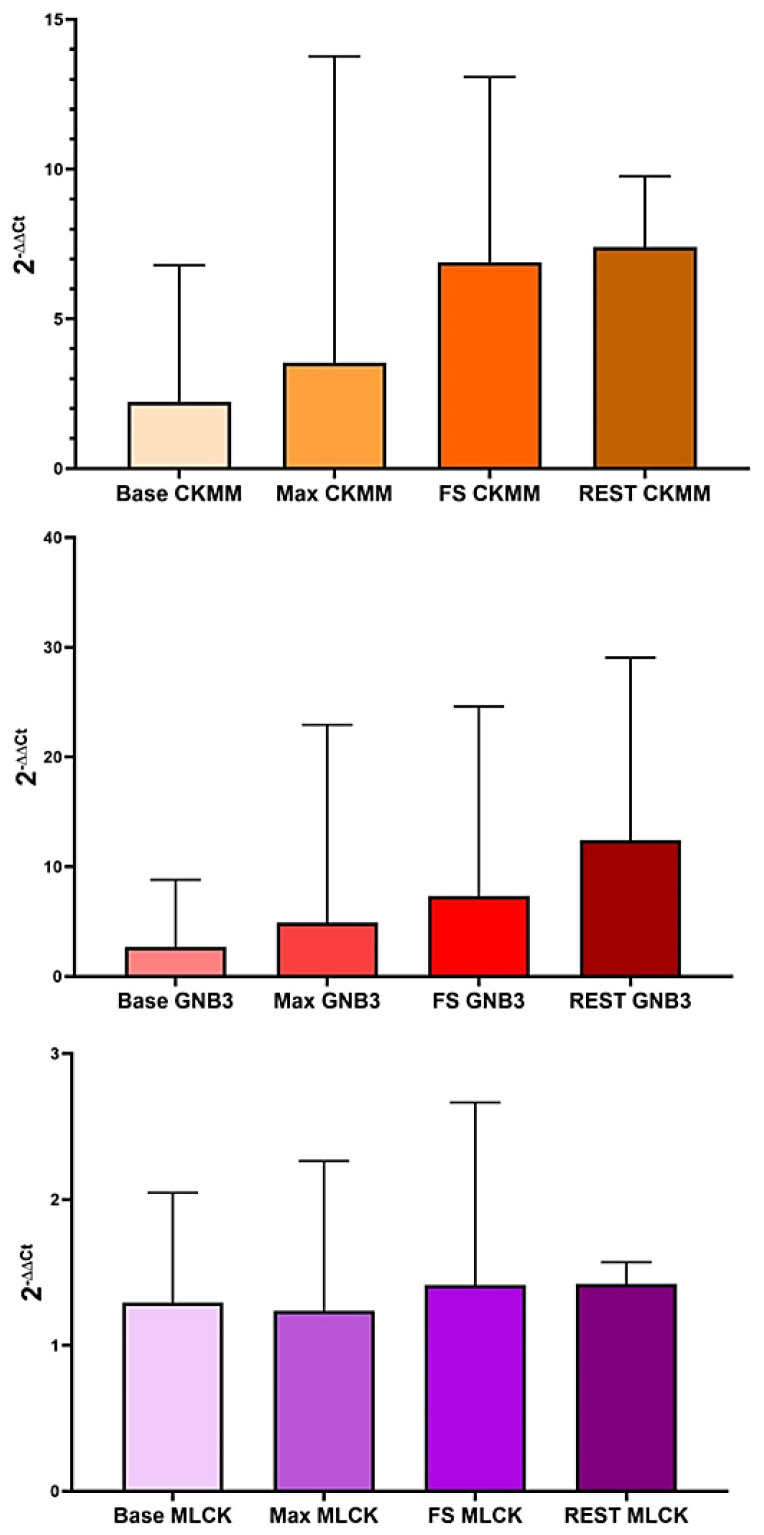
The IL-6, BDNF, and CNTFR expression levels during baseline, after-MVIC, after 50% MVIC, and after-rest conditions.

The result of the repeated measure of ANOVA illustrated that IL-6 did not significantly differ among conditions. Fluctuating expression levels for the IL-6 gene were revealed in the groups. At the baseline, the level of IL-6 transcript was 1.33 ± 1.02. The maximum level of the IL-6 gene was obtained after 50% MVIC (1.87 ± 1.38) (Figure 3).

Although an increased level of the BDNF after 50% MVIC has been observed, the mean SD value of 50% MVIC condition was 13.82 ± 51.13, and there was no significantly different from the baseline (Figure 3).

Similar expression patterns of the CNTRF gene in the groups were observed. The maximal level of the CNTRF was seen after 50% MVIC (2.06 ± 1.55) (Figure 3). This result suggested that the expression state of the CNTFR, ciliary neurotrophic factor receptor, is tissue specific.

**Figure 4:**
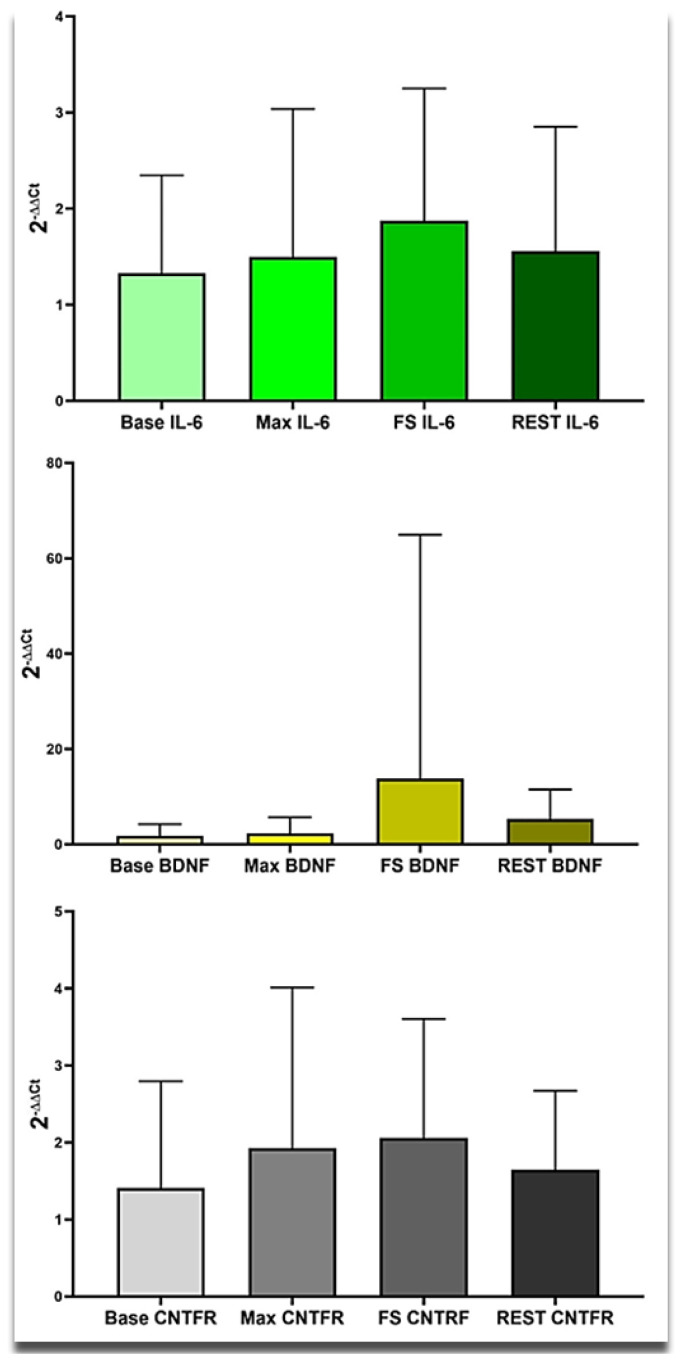
The CKMM, GNB3, and MLCK expression levels during baseline, after-MVIC, after 50% MVIC, and after-rest conditions.

CKMM values steadily increased throughout tests, as seen in Figure 4. Based on the significant result of repeated measures of ANOVA, we decided to compare gene expressions among the load conditions [F_(1.19, 10.73)_ = 10.14, p = .007, η^2^ = .53]. Results demonstrated a borderline significant difference between baseline and after-rest CKMM values. The CKMM expression was also significantly different between the after MVIC and the after-rest values.

An increasing expression level of the GNB3 transcripts was seen during the exercises and rest periods. The maximal level of the GNB3 gene was 12.43 ± 16.61 (Figure 4). This data suggests that the GNB3 continues to be expressed after resting to compensate for oxygen starvation of exercising muscle tissues.

Intriguingly, the MLCK levels were not affected by exercise conditions, as seen in Figure 4. In the baseline condition, the MLCK level was 1.29 ± 0.75 results of the repeated measure of ANOVA illustrated that the MLCK values did not significantly differ among conditions.

## 4. Discussion

The genetic architecture of a given organism undoubtfully contributes to its physical activity. In this context, genetic factors affecting physiological, athletic, and mental performance have become a focus in sports science. Although research in this field has increased in the last few years, the potential role of genetic factors on physical activity remains controversial. In particular, there are relatively few papers describing the influence of genetic factors on the duration and strength of training. Although many genetic polymorphisms affecting sporting activities have been identified in the literature, the relationship between the expression levels of force-related genes and decreased FS acuity has not been fully elucidated. Here we proposed an experimental design that analyzes the expression level of a series of candidate genes during the exposition of elite athletes in a series of standardized training.

We first tested gene expression levels of the IL-6 in various performance conditions, including baseline, after MVIC, after 50% MVIC, and after resting. Present study results indicated that the mRNA level of the IL-6 relatively increased after MVIC and 50% MVIC conditions compared to baseline, whereas it decreased after the resting period. It has been reported that plasma IL-6 level was transiently increased in response to physical exercise conditions, suggesting that this increase is strongly associated with hypertrophic skeletal muscle growth [19]. On the other hand, increased concentrations of IL-6 in actively contracting skeletal muscle fibers after physical activity has been reported [20]. In our qRT-PCR results, the increased IL-6 transcript level during and/or after MVIC suggested that the source of the IL-6 proteins might be skeletal muscle and blood samples.

The ACE coding gene, ACE, is another genetic marker evaluated in this study regarding physical performance. Early studies have reported that insertion/deletion (I/D) polymorphism within the ACE locus is responsible for the variability in plasma ACE levels, and the polymorphism of the ACE might be essential in monitoring hypertension [21]. Previous studies have shown that the I and D alleles are closely associated with endurance and strength gains of elite athletes, respectively [22]. The ACE transcript was transiently increased in our results. After 50% MVIC, the ACE level was higher than that of the baseline group (p < .05). Besides, in the resting period, 15 min from 50% MVIC, the ACE expression was relatively decreased. We did not perform any polymorphism analysis here. However, these results, consistent with a previous study [23], suggested that a surge in the transcript of ACE presumably depends on the strength of performance, but it may also be associated duration of intervals between exercises. The ACTN3 gene encodes a-actinin protein, a structural component of skeletal muscle. A correlation between ACTN3 and sportive activity has been revealed [24]. In our study, the expression level of the ACTN3 was significantly increased after 50% MVIC compared to the baseline. Intriguingly, it was relatively decreased after the resting period. This result suggests that the ACTN3 gene is strongly associated with sportive performance in elite Turkish athletes. However, activation of this gene might occur during the exercise and increase after the %50 MVIC.

A series of polymorphisms within the CKMM locus has been attributed to the performance status of elite athletes [25]. In our qRT-PCR results, although no significant association has been observed between test groups, a relative increase was seen in MVIC and 50% MVIC performance groups and in the resting group after the 50% MVIC. With this finding, we proposed that not only polymorphisms within the CKMM locus but also the expression level of this gene might be associated with the strength of the performance. It has been previously suggested that increased CKMM enzyme expression may involve muscular fatigue in normal circumstances [26]. In our experiments, increased expression of the CKMM transcript in the resting group presumably confirms this scenario.

Recent studies indicated that genetic variants of the MLCK, especially C37885A polymorphisms, may be associated with exercise-dependent muscle damage [8]. However, we observed no obvious difference in the MLCK expression levels between the baseline and the experimental groups. This might be interpreted as the MLCK having no clear effect on our experimental design.

BDNF is a critical growth factor in many aspects. During exercise and at resting status, it is mainly released to circulation from the brain [27]. We found that the level of the BDNF transcript was relatively increased in blood samples of elite athletes after 50% MVIC, and this increment was decreased after the resting period. Consistently to our findings, physical activity causes the synthesis of BDNF in both the brain [28–30], and the periphery [31,32]. Although the present study shows that the expression level of the *BDNF* transcript is relatively increased after 50% MVIC and not after the maximal performance (i.e. 100% MVIC), further studies on a larger scale are needed to clarify which molecular mechanisms may be responsible for this transient activation.

The CNTFR, with its cognate ligand, is essential for neuron survival. Various polymorphisms in the CNTFR locus have been associated with physical activity [33,34]. Furthermore, it has been previously suggested that muscular atrophy and functional loss of muscle caused by denervation and age are also associated with an increased level of the CNTRF transcript in a rat model [35,36]. However, little is known about the expression level of the CNTRF gene during different muscle strength conditions. Here we presented that, although no significant difference was observed, the expression level of this gene was relatively and transiently changed, especially after MVIC and 50% MVIC, as compared to baseline. Indisputably, these alterations in response to physical strength should not be considered atrophy or a functional loss in the skeletal muscle. However, more detailed studies should be performed on polymorphisms of the CNTRF locus and gene expression levels.

The expression level and polymorphisms of the GNB3 gene may be important in regulating some characteristics that affect physical performance, such as cardiac and pulmonary functions, the oxygen transport capacity of the blood, and muscle contraction [37]. This study observed a borderline increase in the GNB3 transcript after MVIC and 50% MVIC compared to the baseline. Interestingly, the expression level of the GNB3 was maximized after rest, most likely because muscle tissues continue to require oxygen during the resting period after exercise.

Although maximal force represents an essential ability in sports, submaximal force accuracy and perception are also important [38]. The present study showed that participants with higher MVIC values had higher force replication errors. The stronger the knee extensors were, the higher the absolute error during the knee extension task. It may mean that those with higher muscle strength have a worse FS. This result could be explained by maximal muscle activity and fatigue caused by exercise. Exercise can disturb proprioception, which has implications for musculoskeletal injuries [39]. Muscle fatigue is not just a matter of peripheral mechanisms accompanying the depletion of muscle energy supplies but includes activation processes at spinal and cortical levels.

## 5. Conclusion

The main finding of this study is that those who develop maximal strength too much may have impaired force acuity. Since maximal strength is significant in many sporting activities, athletes focus on maximal strength development. However, athletes should be aware that this may cause problems in correctly adjusting the level of force at submaximal workloads. ACE and ACTN3 gene expression increased significantly in parallel with increasing force acuity errors. This may be one of the reasons for the deterioration.

In conclusion, we evaluated a series of genes associated with physical exercises. We found that these gene expression fluctuations (i.e. maximization and decreasing) at 50% MVIC and after the rest are likely related to changes in cellular metabolism leading to fatigue.

Time points of gene expression levels during exercise need to be considered. There is not enough information in the literature on how long and at what level most gene expressions respond to exercise. New and important information on when blood samples should be taken has been presented.

## Data Availability

Six authors in the study have approved the use and publication of copyrights by the Turkish Journal of Medical Sciences.
